# Clinical History of a Case of Diphtheritic Metritis, and One of Acute Diabetes

**Published:** 1873-10

**Authors:** John E. Lockridge

**Affiliations:** Augusta County, Virginia


### CLINICAL HISTORY OF A CASE OF DIPHTHERITIC METRITIS, AND ONE OF ACUTE DIABETES.

BY JOHN E. LOCKRIDGE, M. D., OF AUGUSTA COUNTY, VIRGINIA.

Aside from the general interest and instruction in the clinical history of cases, it seems to me that there is some special interest attached to the following two cases; inasmuch as the latter must be very rare, and the former by no means frequent, if we judge from the reported cases. The fatal termination of both should not detract from their interest.

I.

On December 20, 1868, I was called to see Jane Burns, a mulatto woman, about night. On the three-miles journey the messenger, her husband, informed me that his wife was in labor, and that it seemed to be a very slow case, as the one hand and arm had been born the day before: not a very pleasant announcement. The patient was a woman of stalwart frame and herculean strength; she was about 40 years of age, in perfect health, and was the mother of five or six children; she had had no difficulty in her previous labors, except a fashion she had of having premature deliveries—at seven months on several occasions, the mother and child doing well each time; she had been in labor more than 24 hours, with the hand of the child hanging out of the vulva nearly 24 hours; waters evacuated and the pains terrific. I recognized a left hand at once, and the position to be the right cephalo-iliac position of the left shoulder, or left lateral plane of the child's body; the child was dead, of course, and the arm and hand enormously swollen. I will here take the liberty to make a slight diversion. If these lines should chance to fall under the eye of any accoucheur who knows less about the art than I do, I would suggest the following as being the readiest method of making a diagnosis in these cases of arm presentations: just salute the new being with a shake of the hand; this act of friendly recognition can be dexterously and successfully accomplished only with like hands, that is, the palms can only be brought in contact, and the thumbs made to interdigitate by applying the right hand to a right hand, and left to left; and the rule is not impaired by the salutation being posthumous on the part of the child. If it is found that your left hand *fits*, as it were, the little protruding hand, you may know that you have a left position to deal with; and by passing up one or two fingers to ascertain the outlook of the axilla, nothing can be easier than the diagnosis of any case. The axilla in this case looked to the mother's left.

There was nothing left for me but a resort to podalic version; to wait for spontaneous version or evolution would have been futile and absurd; and this operation proved to be one of the most difficult that I have ever performed in obstetrics. I have applied the forceps and delivered from the superior strait in a pelvis measurably contracted in its conjugate diameter, but it was an easy matter in comparison with the operation before me. The enormously swollen condition of the hand and arm, the waters having been evacuated so long, and the womb having been powerfully contracted upon its contents, rendered the introduction of the hand and the seizure of the feet difficult in the extreme.

I succeeded in securing the feet, but before I had time to bring them down a powerful pain came on, which benumbed and crippled my hand so much that I was compelled to let go my hold and recuperate my hand in the folds of the child's body. After resting my hand—the left one—for a minute or so, I completed the version, bringing down the feet, and after considerable delay in delivering the head, the labor was completed by the delivery of the placenta in a reasonable length of time. The child was to full term, and proved to be hydrocephalic to a considerable degree. The patient was not greatly worsted, further thàn a great degree of soreness, and tenderness, on pressure, was complained of.

Dec. 24.—I was re-called, to find my patient in the midst of a terrible attack of the aggregated phlegmasiae, generally denominated puerperal fever. If I be allowed the expression, she was suffering from endo-pelvo-abdominitis. There was not only metro-peritonitis, but para and peri-metritis, salpingitis, and ovaritis—the contents of the pelvic and abdominal cavities were a seething cauldron of inflammation. She was suffering great pain and distress, which was ushered in by a violent chill the day before. I bled her to the tune of 24 oz., and treated her with hot fomentations, and enormous doses of opium for the first few days; and further on, resorted to Dover's powder and quinine and blisters, dressing the blistered surfaces with mercurial and iodine ointments, especially the surface over the left ovary, which, now that the general tympanitic distension had subsided, seemed to be the size of a very large apple.

Jan. 15, 1869.—Under the above general plan of treatment, the disease gradually yielded, until, at this date, she is able to be up, with a good appetite; she had returned to the use of her tobacco pipe, and scarcely any symptoms of disease remained, except an appreciable enlargement of the left ovary. I had reduced the frequency of my visits to 4 or 5 days apart.

Jan. 19.—I was recalled, to find that my patient had had a considerable uterine hemorrhage for the past day or two, and that she was complaining of excessive prostration and pain, confined to the uterus alone, which was swollen and excessively tender to the *touch.* I was led to diagnose diphtheritic endometritis, from the fact that the great frequency of pulse and asthenia were entirely out of proportion to the loss of blood; and besides, I was treating a case of nasal diphtheria within plain view of this house, and about 300 yards from it, in a Northeasterly course, both houses being located in the same ravine, as it were; and a great deal of communication between the two places, both by the doctor and others, rendering the transmission of infection quite likely. I now treated the case with tinct. chloride of iron, quinine, and brandy for 8 or 10 days, with partial success; for whilst the strength and comfort of the patient improved, yet her pulse was very frequent, and there was total loss of appetite and other unfavorable symptoms.

Jan. 26.—On my arrival to-day I was told by the women present that the patient had passed "another after-birth" the day before, and they had saved it for my inspection. Although very putrid, it was plainly a vast diphtheritic membrane, an exact cast of the mucous membrane of the uterus, and in bulk just about filled a pint measure. The patient by this time was able to be up some time, and just before my arrival on the 28th, the patient having been up some time, on again lying upon the bed, immediately expired, the immediate cause of death being, doubtless, cardiac failure from septicæmia or embolism, or both, the uterine mucous membane being favorable now for absorption.

II.

Nov. 14, 1872.—Mr. M. came to my office to consult me about his daughter, aged three years; he said her symptoms were very frequent micturation, languor, &c. A dose of vermifuge had brought away twenty-three worms, some lumbrici and some ascarides. I spoke to him of diabetes, but rather favored the idea of diuresis, with worms in the rectum, as being the probable cause. I gave him some fluid extract of ergot, of which ten or twelve drops were to be given three times a day, and recommended a diet of skimmed milk.

Nov. 18.—Mr. M. returned and requested me to visit his daughter on the next day, as there was no improvement in her case. Suspecting more worms, I directed him to give her another dose of vermifuge that evening, and to follow it next morning with ol. ricini, to insure an operation before my arrival.

Nov. 19.—I visited the patient for the first time. I find that she has slight fever; considerable thirst; a languid, sickly appearance, with a dark circle around her eyes; tongue coated with a heavy brownish yellow fur; mouth clammy; pulse 128 to the minute; bowels obstinately constipated, there being no action from the vermifuge and oil; she was passing about one gallon of clear urine in twenty-four hours; some tenderness on pressure over left lobe of liver, with dullness over a larger space than normal; a disposition to remain all the time on her mother's lap, although plenty strong to run about. She had been passing this large amount of urine for about two weeks; an aunt on the father's side had died recently of something like diabetes. ℞. Hyd. chlorid. mit. pulv. jalap aa ℈ ss m. ft. chart. No. two. s., one to be taken at once, and the other in four hours; continue ergot and skimmed milk. I obtained a specimen of urine, which, being subjected to both Moore's and Trommer's tests, showed a large amount of sugar. I had but a small portion of the urine left, the specific gravity of which I intended to take next morning, but it froze very hard on the vial during the night, and I abandoned the attempt until I could secure another specimen.

Nov. 21.—General symptoms not much changed; some diminution in the quantity of urine, but the amount could not be accurately estimated, as a good deal was passed in the bed; tongue cleaning; bowels well moved—no worms; no enlargement or soreness about the liver; pulse 112; no fever, and skin more moist; less thirst, and tolerable appetite. Continue ergot and skim milk, with some lean meat; this diet to be absolute; ordered flannel next to skin. A specimen of urine obtained showed by the usual tests a less amount of sugar; specific gravity 1,033. This was doubtless a much less specific gravity than the first analysis would have given, as the tests showed much less sugar.

Nov. 23.—Bowels constipated; pulse 130; some heat of skin, and no appetite; great thirst; specific gravity 1,029. Ordered brisk purge and continue ergot, milk, &c., and no other diet. (Four pints of urine in twenty-four hours.)

Nov. 25.—Patient worse; bowels constipated; tongue dry; no appetite; skin very dry, and breathing very laborious; pulse 140; urine half a gallon, and less sugar by Trommer's test; patient extremely weak, and evidently sinking; hardened feces felt through abdominal parietes; fever, &c., Gave enema, and washed away large amount of hardened feces; gave warm bath, and ordered three grains Dover's powder every three hours; milk and wine.

Nov. 25.—Patient rapidly sinking; breathing loud and very labored; skin perfectly dry; urine nearly suppressed; consciousness in abeyance. Resorted to the vapor bath, and continue milk and wine.

Nov. 27.—Patient moribund. No suggestions as to further treatment. The patient died during the night, just nine days from the commencement of the regular treatment, and but little more than three weeks from the time the first symptoms were noticed.

It seems to me that this case presents several points of interest. The case was clearly one of hereditary transmission. The duration of the case would not have been more than a month, even assuming that the parents, ever watchful of the health and welfare of their children, may have overlooked the symptoms for a week or ten days at the beginning. Although of so short duration, yet it ran on through all the usual train of symptoms of chronic affliction; in the start, excessive micturation, thirst and inordinate appetite, progressive emaciation and debility; further on, languor, hepatic engorgement and tenderness from the increased glycogenic function of the organ; the gums swollen and spongy, and an abundant collection of the saccharine material about the bucchal cavity; very dry skin, suppression of urine, coma and stertorous breathing, and death. The prognosis was especially perplexing, for although, at first sight, the objective symptoms showed the case to be very clearly one of diabetes, yet I declined to give an opinion until after the crucial test of analysis; and even after having ascertained that there was a large amount of sugar in the urine, I wished to be very guarded in rendering an opinion.. I frankly told the parents that a few years ago every case of diabetes was looked upon as hopeless, but that recently the prognosis was not quite so grave, as there had been quite a number of well authenticated cases of cure; and that taking into consideration the age of the patient, I had a reasonable degree of hope that I might be able to arrest the malady by a faithful and assiduous application, in turn, of the remedies vaunted of late as curative. But let the worst come to the worst, the end could not certainly be near, as the disease was one, for the most part, of many months, or perhaps year's duration. In this last opinion I was doomed to be wofully disappointed, for in less than one week the glycosurian temple was silent in the tomb, and already the saccharine tide was surging down the red river of life, bartering its sweet but lethean commerce at every citadel of vitality; and although at first there was a seeming amendment under treatment, as shown by a marked diminution, both in the amount of urine and its specific gravity, yet the great centres of life had already suffered too much to recuperate from this illicit and baneful commerce.

*Mount Solon, Va., Aug.* 26, 1873.

---

Ether as an Anæsthetic.—Ether is gradually growing in public favor in England as well as in the United States, as an anæsthetic agent, and is destined sooner or later to displace chloroform. Several articles have lately appeared in the British Medical Journal and other leading medical journals in Europe and the United States, in favor of the use of this agent in preference to chloroform as being much safer, and at the same time quite as good an anæsthetic.—*Canada Lancet.*

